# Letter to the editor concerning “Association of hardware removal with secondary osteonecrosis following femoral neck fractures: a systematic review and meta-analysis”

**DOI:** 10.1186/s13018-023-04495-w

**Published:** 2024-01-08

**Authors:** Wenkai Shao, Ping Wang, Bo Wang, Lin Zou, Yong Feng

**Affiliations:** 1 Department of Orthopedics, Union Hospital, Tongji Medical College, Huazhong University of Science and Technology, Wuhan, China; 2 Department of Rehabilitation, Tongji Medical College, Huazhong University of Science and Technology, Wuhan No. 1 Hospital, Wuhan, China

## Dear Editor,

The article "Association of hardware removal with secondary osteonecrosis following femoral neck fractures: a systematic review and meta-analysis" by Jiang et al. aroused our interest considerably [1]. The authors should be praised for their comprehensive and well-structured meta-analysis, which revealed that hardware removal can lead to a higher occurrence of femoral head osteonecrosis (ONFH) in fracture-healed individuals who received internal fixation for femoral neck fracture. However, the research raises a few insightful queries that will benefit readers with clarifications.

First, only four databases were searched by the authors: PubMed, Embase, Web of Science, and the Cochrane Library. However, there were other more essential electronic databases (including CENTRAL, Medline, ScienceDirect, and clinicaltrials.gov) that could help in collecting more worthwhile research. Also, the search strategy needs to be improved to ensure that few findings are overlooked. Authors are encouraged to utilize a combination of MeSH terms and entry terms.

Second, a random-effects model is more appropriate considering the moderate to high heterogeneity of the pooled data. With a random-effects model, the pooled analysis found no statistically significant difference in the risk of ONFH in the hardware removed group relative to the hardware retained group (OR 0.72, 95% CI: 0.30–1.74, I^2^ 72%), and deletion of Ai did not reverse the outcome (OR 0.51, 95% CI: 0.24–1.05, I^2^ 53%). Based on multivariate logistic regression analysis, the results of the pooled analysis showed no significant differences in ONFH risk compared the two groups (OR 2.05, 95% CI: 0.86–4.88, I^2^ 59%), and the less robust results were shown after sensitivity analysis (OR 1.61, 95% CI: 1.01–2.57, I^2^ 11%).

Finally, despite performing a sensitivity analysis in view of the moderate to high heterogeneity, the authors still fail to explain the source of heterogeneity for several indicators. In addition to the age listed above, gender, various underlying diseases, and type of fracture may all contribute to the study of heterogeneity. Actually, in patients who have had internal fixation, stress shielding may increase the risk of fracture and femoral head necrosis [2, 3]. Zielinski et al. concluded that hardware removal had positive effects on patients' quality of life [4]. Therefore, we should proceed with care when interpreting the results and anticipate further high-quality research.

We extend our sincere gratitude to the authors once more for their exceptional effort and sincerely hope that it will prove beneficial to the readers.

## Data Availability

Not applicable.
